# Prevalence, Patterns and Implications of Chemsex Substance Use Among Gay and Bisexual Men Who Have Sex With Men: A Cohort‐Collaboration Between the Swiss HIV Cohort Study and the SwissPrEPared Study

**DOI:** 10.1002/jia2.70199

**Published:** 2026-09-15

**Authors:** Bashkim Jaha, Andrea Farnham, Roger Kouyos, Jan Fehr, Babette L. Winter, Irene A. Abela, Marcel Stöckle, Bernard Surial, Matteo Reymond, Matthias Cavassini, David Jackson‐Perry, Luigia Elzi, Julia Notter, Benjamin Hampel, Katharina Kusejko

**Affiliations:** ^1^ Division of Infectious Diseases and Hospital Epidemiology University Hospital Zurich Zurich Switzerland; ^2^ Institute of Medical Virology University of Zurich Zurich Switzerland; ^3^ Department of Public & Global Health, Epidemiology, Biostatistics and Prevention Institute University of Zurich Zurich Switzerland; ^4^ Experimental Pharmacopsychology and Psychological Addiction Research, Department of Adult Psychiatry and Psychotherapy, University Hospital of Psychiatry Zurich University of Zurich Zurich Switzerland; ^5^ Division of Infectious Diseases & Hospital Epidemiology, University Hospital Basel University of Basel Basel Switzerland; ^6^ Department of Infectious Diseases, Inselspital, Bern University Hospital University of Bern Bern Switzerland; ^7^ Division of Infectious Diseases, University Hospital Geneva University of Geneva Geneva Switzerland; ^8^ Division of Infectious Diseases, University Hospital Lausanne University of Lausanne Lausanne Switzerland; ^9^ Division of Infectious Diseases Ente Ospedaliero Cantonale Lugano Switzerland; ^10^ University of Geneva and University of Southern Switzerland Lugano Switzerland; ^11^ Division of Infectious Diseases, Infection Prevention and Travel Medicine, Department General Internal Medicine Cantonal Hospital St. Gallen St. Gallen Switzerland

**Keywords:** HIV, longitudinal analysis, PrEP, sexual behaviour, sexually transmitted diseases, substance use

## Abstract

**Introduction:**

Global awareness to chemsex, a specific form of sexualized psychoactive substance (“chems”) use among gay, bisexual and other men who have sex with men (gbMSM), is increasing. We aimed to assess prevalence, patterns and implications of “chems” use on chemsex‐related outcomes.

**Methods:**

We conducted a Swiss‐wide longitudinal study using individual‐level data from April 2019 to December 2024 of adult gbMSM with HIV, enrolled in the Swiss HIV Cohort Study (SHCS), or taking HIV pre‐exposure prophylaxis (PrEP), enrolled in SwissPrEPared. Determinants of “chems” (methamphetamine, new psychoactive stimulants, GHB/GBL) use and clinical implications were analysed using logistic regression. Longitudinal “chems” use patterns were distinguished by rule‐based classification. Point‐prevalences of “chems” use, including COVID‐19 and mpox periods, were analysed using interrupted time series analysis.

**Results:**

We analysed 107,855 follow‐up visits from 13,304 gbMSM. “Chems” were used by 17.2% of gbMSM (21.4% in SwissPrEPared vs. 11.2% in the SHCS). Age below 26 (compared to 26−37, adjusted odds ratio [aOR]: 0.82, 95%‐confidence interval: 0.68−0.96), over 37 (aOR for 38−48: 0.84, 0.75−0.94; aOR for over 48: 0.54, 0.47−0.62) and bisexual orientation showed lower odds (aOR: 0.78, 0.66−0.90), while participation in French‐speaking SwissPrEPared centres (aOR compared to SHCS Eastern Switzerland: 1.41, 1.22−1.64) and sex with non‐steady partners showed higher odds for “chems” use (aOR: 12.44, 8.85−18.17). “Chems” use was associated with depression, inconsistent condom use, high non‐steady partner count and sexually transmitted infections (STIs). Comparing longitudinal use patterns, temporally restricted use, that is, recovering from stable use, reported “chems” use only once, or for a single episode shorter than 1 year, showed the lowest risk for STIs and high partner count, whereas intermittent use showed lower risk only for syphilis. “Chems” use decreased marginally during COVID‐19 and mpox, with a quick return and no difference to levels before COVID‐19.

**Conclusions:**

A large proportion of gbMSM use “chems,” with congruent determinants across both cohorts but heterogeneous impact on sexual and mental health that varies by specific substance and longitudinal use patterns. Further understanding of underlying mechanisms driving this heterogeneity is needed before delivering mechanism‐informed interventions tailored to “chems” use patterns, like STI and depression screening, harm‐reduction counselling and linkage to addiction care.

## 1 Introduction

Recently, there has been increased global awareness in public health and clinical practice of the social phenomenon colloquially termed “chemsex,” particularly prevalent in gay, bisexual and other men who have sex with men (gbMSM) [1, 2, 3]. Chemsex specifically refers to the sexualized substance use (SSU) of specific psychoactive substances immediately preceding or during sexual contact to enhance the sexual experience by gbMSM and taking place in particular settings, contrasting with context‐insensitive recreational substance use [1, 4]. Typical settings are private (anonymous sessions, “chill‐outs,” home parties/“homepas,” “hi‐fun”) or public sex on premise venues (saunas, nightclubs) [4, 5]. However, the dynamic nature of psychoactive substance use with newly emerging “designer drugs” and the variable framing of these diverse settings and practices across economic, social and cultural contexts hinders establishing a consistent definition, which significantly complicates both research and harm‐reduction efforts [4, 5, 6, 7]. Chemsex commonly involves methamphetamine, gamma‐hydroxybutyrate/gamma‐butyrolactone (GHB/GBL), new psychoactive stimulants (NPS), a heterogeneous group of synthetic stimulants (amphetamines and cathinones, e.g. mephedrone) and less commonly ketamine [6, 7].

SSU and chemsex occur globally but more often in large metropolitan cities, with Switzerland ranking among the countries with the highest prevalence [2, 8, 9]. Motivations for engaging in chemsex are diverse and frequently intertwined with complex psychosocial and behavioural factors ranging from experiences of self‐liberation, social belonging and intimacy to more adverse drivers such as (personal) crisis, depression and polysubstance use. These interrelated factors have led to chemsex being conceptualized as a result of syndemic conditions among gbMSM [2, 9, 10, 11, 12].

Advances in HIV antiretroviral therapy (ART) have transformed the sexual health landscape, where treatment as prevention (“U = U”) and pre‐exposure prophylaxis (PrEP) provide reassurance to people with HIV (PWH) and their partners [13, 14]. In this context, changes in sexual behaviour such as increasing condomless anal intercourse have been observed [15]. Chemsex has gained importance as an additional factor shaping sexual networks and health outcomes, particularly through its association with sexually transmitted infections (STIs) [16, 17, 18]. Despite emerging evidence from many qualitative and cross‐sectional studies, there remains a lack of longitudinal and quantitative knowledge on the dynamics of substance use in large gbMSM populations.

We investigate substance use trends with a focus on chemsex substances among gbMSM in Switzerland within two large nationwide longitudinal cohort studies, one enrolling PWH and one enrolling people who are using PrEP. First, we identify sociodemographic, clinical and behavioural characteristics of psychoactive substance use, focusing on differences between these two populations and their clinical implications. Second, we apply rule‐based classification to capture a diversity of individual longitudinal use patterns and understand their impact on clinical and behavioural outcomes. Third, we quantify the impact of the COVID‐19 pandemic and the mpox epidemic on chemsex substance use dynamics within a 5‐year period.

## 2 Methods

### 2.1 The Swiss HIV Cohort Study and the SwissPrEPared Study

The SHCS is an ongoing nationwide, observational cohort study established in 1988 enrolling adult PWH in Switzerland, with an estimated coverage of ∼70% of diagnosed PWH [19]. Participants undergo 6‐monthly study visits, during which detailed clinical and behavioural data, including use of substances, are collected.

SwissPrEPared is a nationwide PrEP programme and nested cohort study in Switzerland launched in 2019 to provide access to PrEP, monitor its use and offer comprehensive care [20]. The study collects clinical data and self‐reported information on PrEP adherence and adverse events, sexual and mental health, and behavioural information including substance use in a sexual setting [20]. Study visits are recommended after 4 weeks of PrEP initiation, followed by 3‐ to 6‐monthly visits depending on the PrEP regimen and sexual behaviour.

The SHCS and the SwissPrEPared study (NCT03893188) were approved by the ethics committees of the participating institutions (BASEC‐Nr. 2023‐02080 and 2018‐02015), and written informed consent was obtained from all participants.

We included data from all cis‐gbMSM with at least one study visit between 1st April 2019 (the start of the SwissPrEPared study) and 31st December 2024.

### 2.2 Variables and Database Harmonization

**FIGURE 1:**
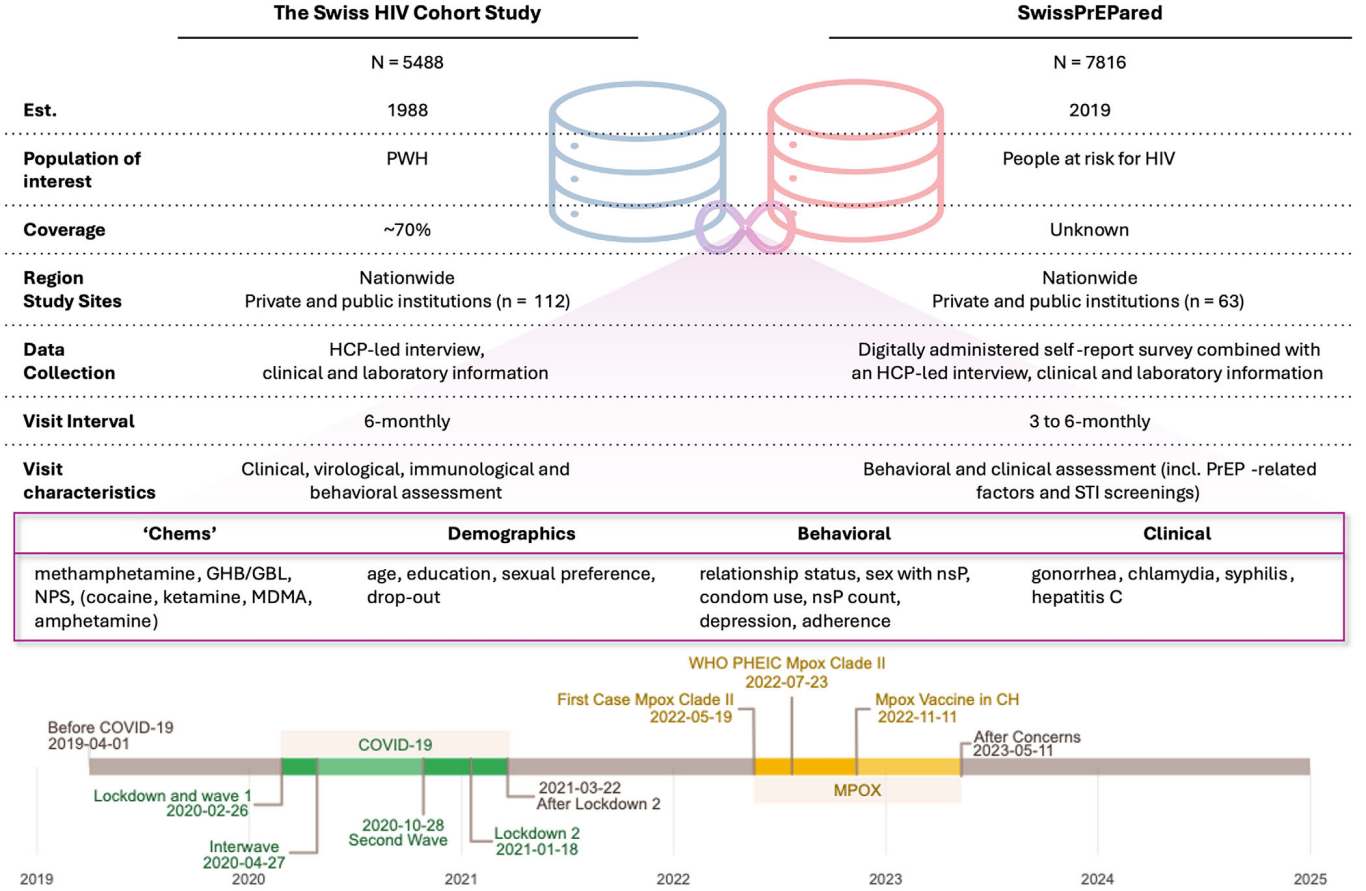
Harmonization of the Swiss HIV Cohort Study and the SwissPrEPared study and study period. The upper table indicates cohort characteristics and differences between the SHCS and the SwissPrEPared Study. For downstream analysis, information on substance use, demographical, behavioural and clinical characteristics were harmonized. The observation period stretched from April 2019 to December 2024, encompassing two major public health events of international concern (COVID‐19 and mpox). CH, Switzerland; COVID‐19, Coronavirus disease 2019; HCP, health‐care provider; HIV, human immunodeficiency virus; nsP, non‐steady partner; PHEIC, public health emergency of international concern; PrEP, pre‐exposure prophylaxis; PWH, people with HIV; STI, sexually transmitted infection.

We used a retrospective data harmonization approach [21]. In brief, we defined demographic, sexual, behavioural, mental health, medical history and substance use‐related variables of interest. Each variable was assessed for harmonization regarding three basic features: semantics, data type (i.e. continuous, ordinal, nominal) and possible responses, including missing value coding. Variables with free text entries were excluded. We categorized corresponding variables from both cohorts as either completely matching, partially matching or not matching and defined them in Table S1. Figure 1 highlights key differences between the cohorts.

### 2.3 Definitions

#### 2.3.1 Substance Use

Psychoactive substance use was assessed with a recall period of 6 months in the SHCS and 3 months in SwissPrEPared, broadly corresponding to visit intervals in each cohort. We defined methamphetamine, GHB/GBL and NPS as “chems,” with NPS combining mephedrone and synthetic stimulants. “Other potentially sex‐enhancing substances” were defined as cocaine, MDMA, ketamine and amphetamine. “Chems” users were defined as participants who reported using the substance at least once during the observation period (also referred to as cumulative use). Analogously, we defined a control population as participants who used no substance apart from alcohol, poppers, cannabis and sedatives/tranquilizers, since these are considered “less harmful” and therapeutic use was not ruled out.

### 2.4 Statistical Analysis

#### 2.4.1 Determinants of “Chems” Use

We used logistic regression to identify associations between cumulative “chems” use and sociodemographic and behavioural variables: age, education, sexual preference, sex with non‐steady partner and a combined cohort‐region variable reflecting study participation. See Figure S1 for the variable selection process.

#### 2.4.2 Influence of “Chems” Use on Clinical and Behavioural Outcomes

In a time‐updated analysis, we assessed whether reporting specific substance use (cases) was associated with higher odds of the following outcomes, compared to the defined controls: feeling depressed, suboptimal adherence (defined as missing more than one pill of ART or PrEP per week for daily regimens or 10 pills for non‐daily regimens), inconsistent condom use, having sex with six or more non‐steady partner within 3 months and a new diagnosis of gonorrhoea, syphilis, chlamydia, or hepatitis C virus (HCV) infection. We constructed generalized linear mixed models (GLMMs) with random intercepts for each participant using the “glmmTMB” package in R. We fitted separate models for each outcome and each psychoactive substance of interest as exposure variable, both uni‐ and multivariable.

#### 2.4.3 Individual Patterns of “Chems” Use

To explore longitudinal patterns of “chems” use, we categorized participants who reported “chems” use at least once and attended more than one visit into seven user profiles with a rule‐based classification (Material S1): reported once, use reported at exactly one visit; consistent users, reporting continuous or near‐continuous (a single gap) use from first to last visit spanning at least 1 year; single episode users, reporting continuous or near‐continuous use up to a period of 1 year or less; sticky starters, reporting no use initially (at least two visits), followed by uninterrupted use up to the last visit; recovered stable users, reporting an early period of use followed by sustained cessation through the remaining (at least four) visits; intermittent stoppers, reporting interspersed “chems” use and no use, ending in a sustained period of no use. Participants whose trajectories did not match any of the above were classified as episodic users, characterized by alternating periods of use and non‐use without a stable terminal cessation pattern. Representation of demographic variables and specific psychoactive substance users was assessed using relative differences (RD) to the included participants for this analysis. We used logistic regression with the aggregated outcome information (i.e. occurrence of above‐defined outcomes at least once during follow‐up) as the dependent variable and the user profiles as exposure variables.

#### 2.4.4 Periods of Public Health Concern

We modelled the course of “chems” use and the effect of the COVID‐19 and mpox epidemic (Figure 1) within an interrupted time series framework [22]. The change over time was modelled through the introduction of breaks in the timeline while modelling abrupt (level) and gradual (slope) changes in the reporting of substance use with generalized estimating equations (GEE) using the R package “geepack” and an exchangeable correlation matrix. We assumed lagged effects of restriction periods, with the optimal lag based on shifted GLMM fits (Material S2).

Across all models, we focused on estimating the magnitude and direction of associations using effect estimates with 95%‐confidence intervals, rather than dichotomized significance testing. As covariates and exposures were based on prior literature, we did not correct for multiple testing, consistent with estimation‐focused observational analyses.

### 2.5 Sensitivity Analysis

We performed several sensitivity analyses. First, we tested whether a suspected selection bias in SwissPrEPared, indicated by a high proportion of PrEP use already before enrolment [20], waned over time, by repeating the analysis of cumulative “chems” use for SwissPrEPared participants registered in 2019, then cumulatively including those enrolled during 2020, 2021, 2022, 2023 and 2024. Second, to understand whether the effect of individual psychoactive substances on the predefined outcomes is affected by polysubstance use, the time‐updated analysis was repeated on the whole dataset—without restriction to controls and specific psychoactive substance users—and included all analysed substances in the multivariable analysis. Third, in the analysis of periods of public health concerns, we repeated the analysis allowing for interaction terms for the time and cohort variables to assess if the cohorts significantly differed from each other in their time trends. Lastly, to assess the accuracy of our collected survey data, we compared quarterly trends with publicly available wastewater measurements on psychoactive substances (methamphetamine, MDMA, ketamine, cocaine, cannabis and amphetamine) measured from 2021 to 2024 [23].

## 3 Results

### 3.1 Study Population

We included 5488 gbMSM in the SHCS and 7816 gbMSM in the SwissPrEPared study with 107,855 follow‐up visits corresponding to 39,086 person‐years of follow‐up (Table 1). Both cohorts enrolled most participants in the Zurich region, but a higher fraction of participants was enrolled in French‐speaking, Western Switzerland for SwissPrEPared (SwissPrEPared vs. SHCS: 32.1% vs. 23.7%). Compared to SHCS participants, gbMSM in SwissPrEPared were younger (median age 33 vs. 50), had a shorter follow‐up time (median 1.6 vs. 5 years) and more often reported sex with non‐steady partners within the last 3 months (97.5% vs. 63.8%). Compared to the SHCS, the fraction of people diagnosed with chlamydia (28.4% vs. 12.3%) or gonorrhoea (28.9% vs. 11.6%) was higher in SwissPrEPared, but the rate of new syphilis was lower (13.1% vs. 16.0%). Of note, 16 SwissPrEPared participants were diagnosed with HIV and were thus excluded from the cohort.

**TABLE 1:** Participant characteristics.

Variable	Total	SHCS	SwissPrEPared	*p*‐value^*^
*N*	13,304	5488	7816	
Born (year), median [IQR]	1980 [1968, 1989]	1969 [1962, 1979]	1986 [1977, 1992]	<0.001
Gender identity, cis‐male	13,304 (100%)	5488 (100%)	7816 (100%)	
Sexual preference				<0.001
homosexual	11,671 (87.7%)	4688 (85.4%)	6983 (89.3%)	
bisexual	1633 (12.3%)	800 (14.6%)	833 (10.7%)	
Nationality or birthplace (region)				<0.001
Switzerland	7498 (56.4%)	3222 (58.7%)	4276 (54.7%)	
Europe	3722 (28.0%)	1462 (26.6%)	2260 (28.9%)	
Americas	1256 (9.4%)	491 (8.9%)	765 (9.8%)	
Other or missing	626 (4.7%)	238 (4.3%)	388 (5.0%)	
Africa	202 (1.5%)	75 (1.4%)	127 (1.6%)	
Highest educational degree				<0.001
University	6007 (45.2%)	1525 (27.8%)	4482 (57.3%)	
Apprenticeship	3536 (26.6%)	2232 (40.7%)	1304 (16.7%)	
Higher education (not university)	2611 (19.6%)	1129 (20.6%)	1482 (19.0%)	
No or compulsory school only	736 (5.5%)	476 (8.7%)	260 (3.3%)	
Other or missing	414 (3.1%)	126 (2.3%)	288 (3.7%)	
Enrolment date, median [IQR]	2020 [2012, 2022]	2010 [2002, 2016]	2022 [2020, 2023]	<0.001
Centre of registration				<0.001
Eastern Switzerland	9493 (71.4%)	4189 (76.3%)	5304 (67.9%)	
Western Switzerland	3811 (28.6%)	1299 (23.7%)	2512 (32.1%)	
Follow‐up time (years), median [IQR]	3.07 [0.98, 4.99]	5.04 [3.85, 5.31]	1.6 [0.48, 3.43]	<0.001
Ever been in a stable partnership	8163 (61.4%)	3954 (72.0%)	4209 (53.9%)	<0.001
Ever had occasional sex	11,142 (83.7%)	3503 (63.8%)	7639 (97.7%)	<0.001
Ever reported ≥6 occasional partners	6256 (47.0%)	1049 (19.1%)	5207 (66.6%)	<0.001
Condom use with non‐steady partners				<0.001
≥1 never	6581 (49.5%)	1989 (36.2%)	4592 (58.8%)	
≥1 inconsistent	3215 (24.2%)	860 (15.7%)	2355 (30.1%)	
no sex with non‐steady partners	1942 (14.6%)	1767 (32.2%)	175 (2.2%)	
always	791 (5.9%)	545 (9.9%)	246 (3.1%)	
Missing/refused to answer	775 (5.8%)	327 (6.0%)	448 (5.7%)	
Ever felt depressed/down	7225 (54.3%)	1714 (31.2%)	5511 (70.5%)	<0.001
Ever had suboptimal adherence	2411 (18.1%)	851 (15.5%)	1560 (20.0%)	<0.001
Gonorrhoea diagnosis	2890 (21.7%)	635 (11.6%)	2255 (28.9%)	<0.001
Chlamydia diagnosis	2895 (21.8%)	675 (12.3%)	2220 (28.4%)	<0.001
Syphilis diagnosis	1896 (14.3%)	876 (16.0%)	1020 (13.1%)	<0.001
Hepatitis C virus diagnosis	48 (0.4%)	16 (0.3%)	32 (0.4%)	0.332
Used other sex‐enhancing substances	3503 (26.3%)	1081 (19.7%)	2422 (31.0%)	<0.001
Used chemsex substances	2289 (17.2%)	615 (11.2%)	1674 (21.4%)	<0.001

*Note*: Variables related to follow‐up refer to the observation period only and do not regard prior participant history for SHCS participants. Information on sexually transmitted infections was only regarded with a margin of 70 days prior and 20 days after the corresponding follow‐up visit.

Abbreviation: IQR, interquartile range.

* *p*‐values were derived from a two‐sample *t*‐test for continuous and Pearson's chi‐squared for categorical variables.

### 3.2 Cumulative Substance Use and Associated Sociodemographic and Behavioural Factors

**FIGURE 2:**
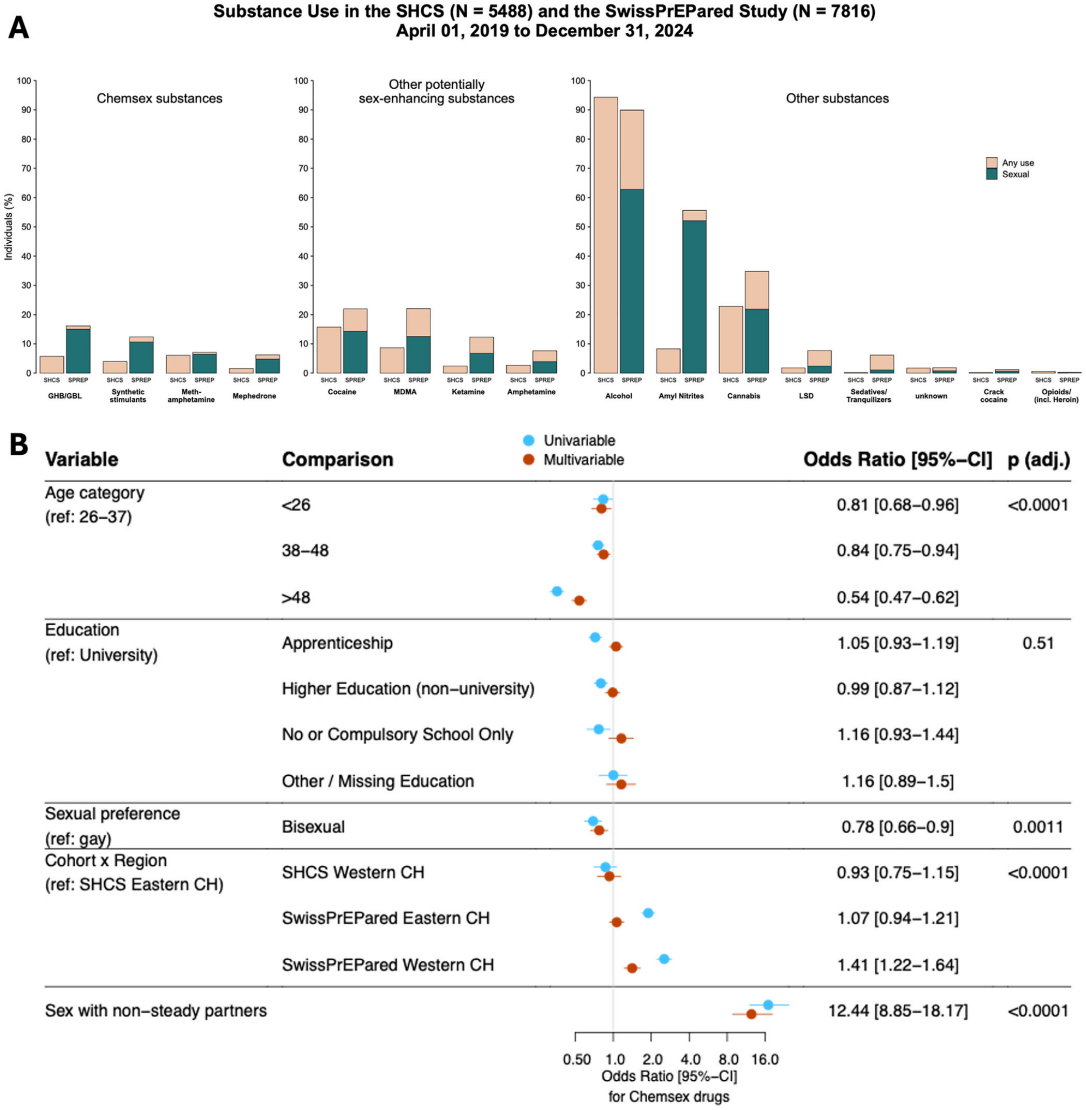
Substance use among gbMSM. (A) Cumulative substance use showed different levels of use, with more SwissPrEPared participants encountering substances. (B) Sociodemographic and behavioural predictors of “chems” use. Results from uni‐ and multivariable logistic regression to discern predictors for reporting chemsex substance use at least once during the observation periods are shown in a coefficient plot. Age at registration was categorized after inspecting its distribution with respect to “chems” use. Age below 26 and over 48 and bisexual orientation showed lower adjusted odds ratios (aOR) for reporting chemsex substance use, while reporting sex with non‐steady partners and participants in SwissPrEPared, particularly from the French‐speaking, Western Swiss region, showed higher aOR. CH, Switzerland; SHCS, Swiss HIV Cohort Study.

In total, 2289/13,304 (17.2%) individuals reported using “chems” at least once during the observation period (21.4% in SwissPrEPared vs. 11.2% in the SHCS, Figure 2A). Specifically, the use of NPS and GHB/GBL was substantially higher in SwissPrEPared compared to the SHCS (15.3% and 16.2% vs. 8.2% and 5.7%, respectively), but methamphetamine use was similar (6.5% vs. 6.1%). Almost all SwissPrEPared participants reporting “chems” use also reported to have used them at least once in a sexual setting. Except for cocaine, cannabis and alcohol, the proportion of people who used other psychoactive substances in the SHCS was consistently less than half the corresponding proportion in SwissPrEPared.

Higher age at baseline (aOR for >48 compared to 26−37 = 0.54, 95% confidence interval [CI] 0.47–0.62) and bisexual partner preference (aOR = 0.78, 0.66–0.90) showed reduced odds (Figure 2B), while having sex with non‐steady partners showed increased odds for “chems” use (aOR = 12.44, 8.85–18.17). SwissPrEPared participants from Western Switzerland showed higher odds (aOR = 1.41, 1.22–1.64) for using “chems” compared to SHCS participants from Eastern Switzerland. In separate analyses for the three “chems,” we found pronounced regional differences (Figure S2). GbMSM with HIV in Eastern Switzerland showed increased odds of using methamphetamine, while NPS are more likely to be used by Western Swiss PrEP‐users (aOR = 1.69, 1.44–1.98) and GHB/GBL by PrEP‐users in general.

In a sensitivity analysis, we progressively extended the inclusion cut‐off (end of each calendar year) for SwissPrEPared and observed evidence for a larger effect for the cohort at the beginning (aOR for SwissPrEPared vs. SHCS in 2019: 2.14, 1.79–2.55) than at the end of the period (aOR in 2024: 1.16, 1.04−1.31; Figure S3).

### 3.3 “Chems” Use Comes With Health Implications

Depression showed a strong association with methamphetamine use (aOR = 2.89, 2.39−3.50), similar for other stimulants such as amphetamine and NPS; however, less strong for GHB/GBL and other potentially sex‐enhancing substances (Figure S4). Suboptimal adherence was associated with amphetamine (aOR = 2.52, 1.22−5.28) and methamphetamine use (aOR = 3.02, 1.75−5.22). The difference in association between “chems” and other potentially sex‐enhancing substances was most pronounced for all three STIs, particularly for gonorrhoea (aOR for “chems” = 3.18, 2.87−3.52; aOR for other potentially sex‐enhancing substances = 2.10, 1.92−2.31). Similarly, inconsistent condom use and high non‐steady partner count showed strong association with “chems” use. HCV infection was associated with use of any of the three “chems” (GHB/GBL, methamphetamine, NPS).

In the sensitivity analysis, we investigated if including all the analysed substances in the multivariable analysis led to attenuated effects of substances which are typically co‐used. Univariable results remained similar, but adjusted ORs across all outcomes were strongly reduced, particularly for amphetamine and ketamine, indicating that the associations are partly explained by using other psychoactive substances. This was less pronounced for MDMA and cocaine (Figure S4). Methamphetamine showed mixed results, with a stable association with depression and syphilis but attenuated or absent associations for other outcomes. Interestingly, HCV infection showed persistent association with NPS but also for amphetamine use. These findings highlight the complexity of polysubstance use.

### 3.4 Once “Chems” ≠ Always “Chems”

**FIGURE 3:**
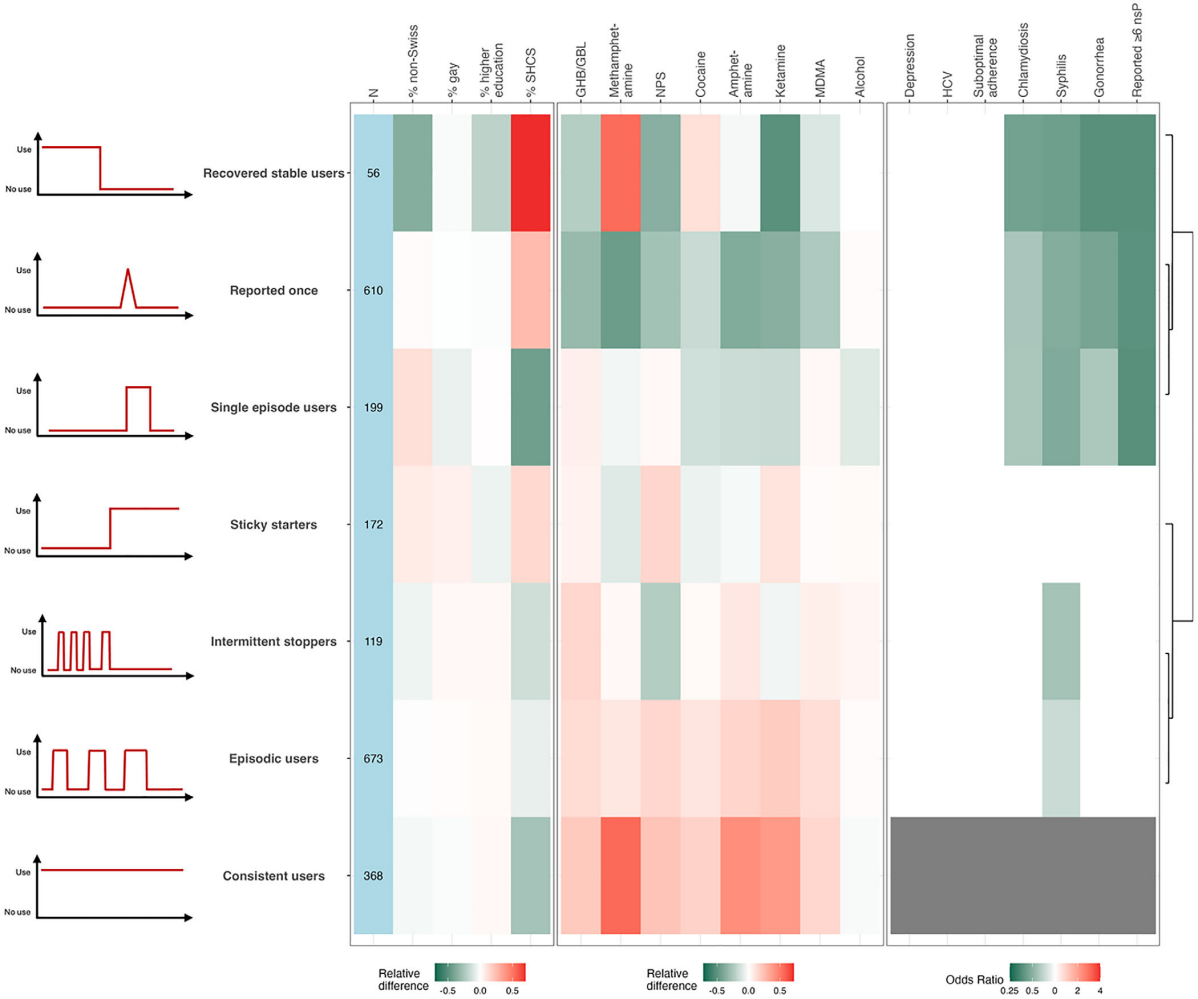
Longitudinal chemsex substance use profiles, demographic variables, key substances used and association with predefined behavioural and clinical outcomes. Trajectories (left‐side) illustrate the exhibited longitudinal reporting pattern for “chems” defined as GHB/GBL, methamphetamine and new psychoactive stimulants. A relative difference was calculated for each demographic variable to assess over/underrepresentation ($\frac{P(X|use\;pattern)\;-\;P(X)}{P(X)})$. For the substances (middle panel), the proportions of participants reporting use of the respective substance at least once during follow‐up and relative difference were calculated consistently. The right heatmap panel visualizes the point estimates for the odds ratio derived from multivariable logistic regression for each use pattern compared to consistent users. The adjustment set of covariables for multivariable analysis included age, nationality, sexual preference, cohort and year of first visit. The user profiles are presented in order resulting from hierarchically clustering the estimates from multivariable logistic regression (dendrogram on the right). HCV, hepatitis C virus; NPS, new psychoactive stimulants; nsP, non‐steady partners; SHCS, Swiss HIV Cohort Study.

We distinguished seven longitudinal “chems” use patterns (Figure 3), with “episodic use” (*n* = 673), “reported once” (*n* = 610) and “consistent use” (*n* = 368) being the most frequent patterns. SHCS participants were overrepresented within “recovered stable users” (RD 0.70) and underrepresented among “single episode use” (RD −0.43) and “consistent users” (RD −0.27). “Chems” and potentially sex‐enhancing substance users were overrepresented among “consistent users.” In the multivariable regression analysis, “consistent users” were more likely to be diagnosed with chlamydia, syphilis or gonorrhoea, and more likely to report having sex with over six non‐steady partners compared to “recovered stable users,” “reported once” and “single episode users.” Further, we found reduced odds of syphilis for “episodic users” and those who stopped after an intermittent use pattern compared with “consistent users.” We found no evidence for differences in depression prevalence, new HCV infection and the reporting of suboptimal adherence between the use patterns.

### 3.5 “Chems” Use During Periods of Public Health Concerns

**FIGURE 4:**
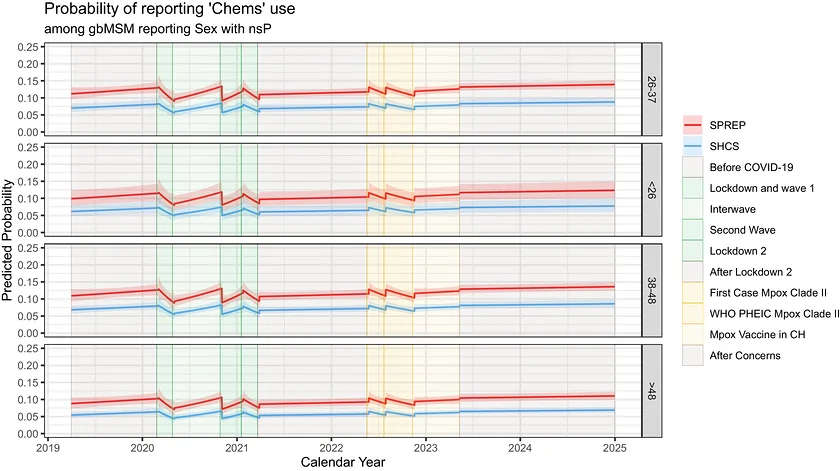
Fitted population‐averaged probabilities of “chems” use derived from a multivariable GEE model for gbMSM reporting sex with non‐steady partners. Time frames outside periods of public health concerns are shaded in grey‐brown. Periods affected by the COVID‐19 pandemic are shown in green, and mpox epidemic periods in yellow. The lighter shade for both public health concern periods represent times without restrictions or recommendations, that is where we expected a lower impact on “chems” use. CH, Switzerland; COVID‐19, Coronavirus disease 2019; gbMSM, gay and bisexual men who have sex with men; nsP, non‐steady partners; PHEIC, public health emergency of international concern; SHCS, Swiss HIV Cohort Study; SPREP, SwissPrEPared.

During the COVID‐19 crisis compared to “before COVID‐19,” we observed lower odds of “chems” use at the onset (aOR = 0.69, 0.56−0.84), but an increasing trend during the “interwave” period (aOR per 30 days = 1.05, 1.00−1.09; Figures 4 and S5). “After COVID‐19 lockdown 2” in 2021, “chems” use remained lower compared to “before COVID‐19” (aOR = 0.68, 0.48−0.98), with no evidence for difference in trends/slopes to “before COVID‐19” (*p*_trend_ = 0.4). After declaration of mpox as a public health emergency of international concern, odds for “chems” use were slightly lower than “before COVID‐19” (aOR = 0.92, 0.87−0.98). Neither abrupt nor gradual changes were found before the declaration and after the mpox vaccine was made available in Switzerland. We found no significant trend in “chems” use among gbMSM “before COVID‐19” and “after concerns” in our GEE model (“before COVID‐19”: *p*_trend_ = 0.13; “after concerns”: *p*_trend_ = 0.25; Figure S6).

Notably, the conditional model showed similar but slightly larger effects of public health concerns, though with no evidence for a difference between cohorts (Figure S6; *p* = 0.11), contrasting population‐averaged findings (aOR for SHCS vs. SwissPrEPared = 0.60, 0.52–0.69). The large intraclass correlation coefficient (ICC = 0.96) and variance of the random intercept (σ^2^ = 86) indicate a large heterogeneity within the population that cannot be explained by cohort membership.

Quarterly trends parallelize with the available wastewater reports on metabolite estimates, particularly for methamphetamine and MDMA (Figure S7). This supports that our survey data accurately depict trends found by independent analyses; however, with a variable delay depending on the analysed psychoactive substance.

## 4 Discussion

In this study, we investigated the longitudinal “chems” use in a large population of gbMSM within two distinct cohorts in Switzerland.

Substance use was highly associated with demographic and behavioural factors, particularly age between 26 and 37, sexual activity and homosexual orientation were consistent predictors of cumulative “chems” use—in line with prior literature [2]. We initially found strong differences between gbMSM enrolled in the SHCS and SwissPrEPared, with an approximately two‐fold higher overall reported consumption of the analysed psychoactive substances in SwissPrEPared as compared to the SHCS. This difference is no surprise, given that the SHCS is estimated to have enrolled ∼65% of all Swiss people diagnosed with HIV since its launch, with especially good representativeness of gbMSM [24], while SwissPrEPared, particularly during the early phase, was rather addressing Swiss gbMSM at high risk for HIV related to sexual behaviours [20]. After adjusting for sociodemographic and behavioural variables, we found little difference between the cohorts, likely attributed to regional differences, which contrasts with prior publications showing higher prevalence of “chems” use among gbMSM with HIV [2]. A possible explanation is that our study covers a large gbMSM population in Switzerland after the systematic rollout of PrEP, whereas most studies in the field were conducted during or before its broad implementation.

Psychoactive substance use was significantly associated with increased odds for STIs, which were most pronounced for gonorrhoea and “chems” use. The two‐ to three‐fold increase in odds of gonorrhoea among “chems” users, but the less pronounced increased risk for chlamydia infection also aligns with previous studies [18, 25]. Transmission of STIs remains understudied, with no study investigating the impact of “chems” on mucosal barriers, for example through exsiccation aggravated by prolonged or frequent chemsex. Such mechanisms’ impact is likely small compared to behavioural factors. Further, the association of certain “chems” with a high number of non‐steady partners, condomless sex, suboptimal adherence and depression aligns with findings in the SHCS and numerous other studies [26, 27]. The difference in magnitude of the association with the specific “chems” likely reflects varying motives and risks associated with substance use, among which particularly NPS demand further attention [11].

Longitudinal “chems” use patterns were heterogeneous, with “episodic use,” “reporting once” and “consistent use” being most frequent. The overrepresentation of methamphetamine users among “consistent users” and underrepresentation among those reporting “chems” use once may reflect not only its high addiction potential but also a complex interplay of the individual's identity and preferences with its embedding within specific subcultures, with stigma potentially acting as a barrier to occasional use [2, 9, 28].

The predominance of “episodic” rather than “consistent use” complicates framings of SSU as inherently or uniformly problematic. The heterogeneity in frequency, specific substance and longitudinal use accords with the notion of distinguishing occasional, recreational SSU from sustained or dependent patterns rather than treating “chems” use as a single high‐risk behaviour. However, the episodic category should not be read uncritically. We hypothesize that this pattern comprises presumptively recreational intermittent use, exhibited by gbMSM participating in reccurring events (e.g. circuit parties, “Pride”), alongside longer stretches of persistent use or abstinence/non‐use across multiple consecutive visits. Particularly, the subgroup with predominant use (*n* = 269) may in part reflect repeated unsuccessful attempts of abstinence, a pattern partly consistent with problematic or dependence‐associated use. Since “chems” induce a variety of desired effects alongside their addictive potential [11, 28], their use likely intersects among use patterns and subgroups of gbMSM, spanning low‐risk, event‐driven use through to use that individuals are struggling to reduce.

This heterogeneity was also reflected in sexual health outcomes. Consistent or recurring use was associated with higher odds of STI diagnosis and high non‐steady partner count. Compared to “consistent users,” “intermittent stoppers” and “episodic users” had lower odds of syphilis diagnosis. Similarly, “recovered stable users,” “single episode users” and those “reporting once” had lower odds for all three STIs. This suggests that psychoactive substance use patterns are linked to formation and consolidation of social and sexual networks which sustain STI transmission [12].

We found only minimal impact of periods of public health concerns on “chems” use over time, with relatively stable prevalence levels among gbMSM having sex with non‐steady partners of around 9% in the SHCS and 15% in SwissPrEPared. Although SwissPrEPared participants reported higher use on a population‐level, our conditional model showed that “chems” use is longitudinally clustered within individuals and suggest that the variability is independent of cohort. Prior studies and wastewater reports from the EU Drugs Agency (EUDA) indicate that “chems” use in Switzerland started around 2015 [26, 29]. A comparable trend was found in Australia and Canada [30, 31], though no clear explanation has emerged. In parallel, LGBT visibility on social media (Twitter) has risen [32]. “Chems” and the sex parties where they are consumed are typically marketed through social media and other online platforms [1]. Globalization and technological advances plausibly catalysed the dissemination of “chems” and “chemsex,” comparable to findings of condomless sex with non‐steady partners in the SHCS [15]. These parallel developments suggest a complex interplay between advances in HIV prevention, syndemic conditions and chemsex practices, with insufficiently understood underlying dynamics.

Our study exhibits several strengths and limitations. First, reliance on self‐reported sensitive information may introduce social desirability bias—most likely less in SwissPrEPared through private online data collection. However, our sensitivity analysis supports consistency between cohorts (over time), despite different data collection methods. Second, the absence of biomarkers and external data sources from the broader gbMSM population limits triangulation and quantification of the potential extent of underreporting of “chems” use. However, the trends in our analyses aligning with independent wastewater reports support representability of the cohorts in capturing substance use (Figure S7). Third, surveillance data from Zurich's drug checking service reveal increased harm reduction engagement [33], but low substance concordance, particularly for mephedrone/4‐MMC, suggests incomplete substance and risk awareness or deliberate substitution (e.g. other cathinones). To mitigate the potential underestimation, we aggregated synthetic stimulants and mephedrone as NPS. Fourth, as we did not adjust for multiple testing, associations with confidence intervals near the null should be regarded as hypothesis‐generating. Finally, we focused on public health implications of “chems” use in general and did not further investigate injection practices and potential benefits of self‐expression, social belonging or dyadic chemsex.

## 5 Conclusions

Our study showed stable but prevailing “chems” use in a large Swiss study population of gbMSM with and without HIV. We showed that “chems” use is associated with sex with non‐steady partners, high partner count, inconsistent condom use, depression and STIs, which supports that “chems” are mainly used for chemsex by a heterogeneous population of gbMSM. Surveillance of “chems,” especially cathinones, due to unpredictable short‐ and long‐term harms, demands intensification nationally, including extended wastewater monitoring and active surveillance of “designer drug” dynamics to better inform the community. Interventions, such as routine STI screening, depression screening and referral, harm‐reduction counselling, drug‐checking services and linkage to addiction care, should be systematically integrated into HIV/PrEP services and online outreach directed towards gbMSM. However, the heterogeneity of use patterns and their incompletely characterized underlying mechanisms demand further research before more tailored, mechanism‐informed interventions can be appropriately designed and delivered.

## Author Contributions

BJ, AF, RK, BLW, BH and KK conceived the study. BJ developed the methodology, performed the software implementation, conducted the investigation, curated the data, carried out the formal analyses and created the visualizations. BH and KK validated the analyses. AF, JF, IAA, MS, BS, MR, MC, DJ‐P, LE, JN, BH and KK provided essential resources. KK supervised the project, administered the project and acquired funding. BJ and KK drafted the original manuscript. AF, RK, JF, BLW, IAA, MS, BS, MR, MC, DJ‐P, LE, JN, BH and KK critically reviewed and edited the manuscript. All authors have read and approved the final manuscript.

## Members of the Swiss HIV Cohort Study

Abela IA, Aebi‐Popp K, Anagnostopoulos A, Bernasconi E, Boyd A, Braun DL, Bucher HC, Calmy A, Cavassini M (Chairman of the Clinical and Laboratory Committee), Chaudron SE (Head of Data Centre), Ciuffi A, Dollenmaier G, Egger M, Elzi L, Fehr JS, Fellay J, Frigerio Malossa S, Fux CA, Günthard HF, Hachfeld A, Haerry DHU (deputy of “Positive Council”), Hasse B, Hoffmann M, Huber M, Jackson‐Perry D (patient representatives), Kahlert CR, Kaufmann D, Keiser O, Kouyos RD, Kovari H, Kusejko K, Labhardt ND, Leuzinger K, Marzolini C, Metzner KJ, Müller N, Nemeth J, Nicca D, Notter J, Paioni P (Chairman of the Mother & Child Substudy), Perreau M, Rauch A (President of the SHCS), Salazar‐Vizcaya LP, Schmid P, Segeral O, Speck RF, Stöckle M, Surial B, Tarr PE, Trkola A, Wandeler G (Chairman of the Scientific Board), Weisser M, Yerly S.

## Funding

This study was supported by the Swiss National Science Foundation (grant #218408 to KK) and developed within the framework of the Swiss HIV Cohort Study, which is supported by the Swiss National Science Foundation (grant #33FI‐0_229621), and SwissPrEPared, which is supported by the Swiss Federal Office of Public Health (order number 142006945/332.3‐54/20). The data in the SHCS are gathered by the five Swiss University Hospitals, two Cantonal Hospitals, affiliated hospitals and private physicians. The data in SwissPrEPared, in addition to the centres named above, are gathered by the 85 SwissPrEPared centres, including hospitals, community health centres (“Checkpoints” and PROFA), primary and specialized physicians like dermatologists/venerologists, and addiction/substance use specialists.

## Conflicts of Interest

IA's institution received grants from the Swiss HIV Cohort Study, the University of Zurich and travel grants from Gilead Sciences and Biotest. BS's institution reports financial support for travel grants from Gilead Sciences and ViiV Healthcare, and for advisory boards from Gilead Sciences and MSD. MC's institution received research grants from Gilead and MSD and expert opinion fees from Gilead, MSD and ViiV. MC's institution received travel grants from Gilead and MSD. JF's institution received project grants from Gilead Sciences, MSD and ViiV Healthcare. There is no conflict of interest to declare by the other authors (BJ, AF, RK, BLW, MS, MR, DJ‐P, LE, BH and KK).

## Supporting information




**Supporting File**: jia270199‐sup‐0001‐SuppMat.docx

**Supporting File**: jia270199‐sup‐0002‐tableS1.xlsx

## Data Availability

The individual‐level datasets generated or analysed during the current study do not fulfil the requirements for open data access: (1) the SHCS’ informed consent states that sharing data outside the SHCS network is only permitted for specific studies on HIV infection and its complications, and to researchers who have signed an agreement detailing the use of the data and biological samples; and (2) the data is too dense and comprehensive to preserve patient privacy in persons living with HIV. According to Swiss law, data cannot be shared if individuals have not agreed or the data are too sensitive to share. SwissPrEPared obeys the same national regulations. Investigators with a request for selected data should send a proposal to the respective SHCS (www.shcs.ch/contact) or SwissPrEPared address (https://www.swissprepared.ch/en/contact/). The provision of data will be considered by the Scientific Boards of the SHCS and SwissPrEPared, the study team, and is subject to Swiss legal and ethical regulations, and is outlined in a material and data transfer agreement.
