# Supplementary material for: Prevalence, Patterns and Implications of Chemsex Substance Use Among Gay and Bisexual Men Who Have Sex With Men: A Cohort‐Collaboration Between the Swiss HIV Cohort Study and the SwissPrEPared Study
Source: J Int AIDS Soc. 2026 Sep 15;29(9):e70199. doi: 10.1002/jia2.70199 (PMC13575759; doi:10.1002/jia2.70199)
Supplement: Supplementary file 1 [file JIA2-29-e70199-s002.docx]

**Supplementary Material** to

Prevalence, Patterns, and Implications of Chemsex Substance Use among Gay and Bisexual Men Who Have Sex with Men: A Cohort-Collaboration between the Swiss HIV Cohort Study and the SwissPrEPared Study

Bashkim Jaha^1,2^, Andrea Farnham^3^, Roger Kouyos^1,2^, Jan Fehr^3^, Babette L. Winter^3,4^, Irene Abela^1,2^, Marcel Stöckle^5^, Bernard Surial^6^, Matteo Reymond^7^, Matthias Cavassini^8^, David Jackson-Perry^8^, Luigia Elzi^9,10^, Julia Notter^11^, Benjamin Hampel^3^, Katharina Kusejko^1,2*^

^1^Division of Infectious Diseases and Hospital Epidemiology, University Hospital Zurich, Zurich, Switzerland

^2^Institute of Medical Virology, University of Zurich, Zurich, Switzerland

^3^Department of Public & Global Health, Epidemiology, Biostatistics and Prevention Institute, University of Zurich

^4^Experimental Pharmacopsychology and Psychological Addiction Research, Department of Adult Psychiatry and Psychotherapy, University Hospital of Psychiatry Zurich, University of Zurich

^5^Division of Infectious Diseases & Hospital Epidemiology, University Hospital Basel, University of Basel, Basel, Switzerland

^6^Department of Infectious Diseases, Inselspital, Bern University Hospital, University of Bern, Bern, Switzerland

^7^Division of Infectious Diseases, University Hospital Geneva, University of Geneva, Geneva, Switzerland

^8^Division of Infectious Diseases, University Hospital Lausanne, University of Lausanne, Lausanne, Switzerland

^9^Division of Infectious Diseases, Ente Ospedaliero Cantonale, Lugano, Switzerland

^10^University of Geneva and University of Southern Switzerland, Lugano, Switzerland

^11^Cantonal Hospital St. Gallen, Division of Infectious Diseases, Infection Prevention and Travel Medicine, Department General Internal Medicine, St. Gallen, Switzerland

Table S1 Harmonization Table

Figure S1 Separate logistic regression results for sociodemographic and behavioural characteristics associated with cumulative ‘chems’ use

Material S1 Rule-based Use Pattern Recognition Algorithm

Material S2 Interrupted Time Series Model

Figure S2 Predictors of encountering Methamphetamine, GHB/GBL and NPS substance use in the cohorts

Figure S3 Logistic regression results after progressively extending the inclusion cut-off (by end of each calendar year) for SwissPrEPared participants

Figure S4 Regression coefficients derived from (random intercept) GLMMs to analyse the association of the use of specific substance on specified time-updated outcomes

Figure S5 Average prevalence of chemsex substance use (30-day left-aligned moving average)

Figure S6 Comparison of regression coefficients from a generalized estimating equation (GEE) and generalized linear mixed model (GLMM) for chems use over time using and interrupted time series framework

Figure S7 European Union drugs agency (EUDA) wastewater report data from Switzerland

**Figure S1 Separate logistic regression results for sociodemographic and behavioural characteristics associated with cumulative ‘chems’ use**. The separate regression analysis was used to guide variable selection. Non-overlapping 95% confidence intervals for the adjusted odds ratios were observed for being older than 45 and sex with nsP, and Western Switzerland region of enrolment. Age and sex with nsP showed the same qualitative effect on ‘chems’ use, however, this was not guaranteed for the region, hence, we included an interaction term for the cohort with the region in our final model.

| **Material S1 Rule-based Use Pattern Recognition Algorithm** | | | | |
| --- | --- | --- | --- | --- |
|  | | | | |
| Construct binary sequence of responses by concatenating | | | | |
| Evaluate sequentially the following conditions using regular expressions and assign accordingly: | | | | |
|  | *Condition* | |  | *Category* |
|  | n_visits == 1 | | 🡪 | Single visit |
|  | traj matches "^0+$" | | 🡪 | Never users |
|  | count_1 == 1 | | 🡪 | Reported once |
|  | traj matches "^1+$" AND t_episode > 365d | | 🡪 | Consistent users |
|  | traj matches "^1+$" AND t_episode <= 365d | | 🡪 | Single episode users |
|  | count_0 == 1 AND count_1 > 3 AND t_episode > 365d | | 🡪 | Consistent users |
|  | count_0 == 1 AND count_1 > 3 AND t_episode <= 365d | | 🡪 | Single episode users |
|  | traj matches "^0+1+$" | | 🡪 | Sticky starters |
|  | traj matches "^0+1{2,}0*$" AND t_episode <= 365d | | 🡪 | Single episode users |
|  | traj matches "^0+1{2,}0*$" AND t_episode > 365d | | 🡪 | Consistent users |
|  | traj matches "^1{2,}0{4,}$" | | 🡪 | Recovered stable users |
|  | traj ends with "0000" | | 🡪 | Intermittent stoppers |
|  |  | *Else* | 🡪 | Episodic users |

The algorithm depends on only five variables. First, a binary response sequence is constructed – the trajectory – by concatenating the chronologically arranged ‘chems’ use responses (e.g., 0010111111). We then calculated the total number of visits (n_visits), the number of positive responses (count_1), and negative responses (count_0). Further, we calculated the episode length (t_episode) in calendar days for each sequence of contiguous positive responses (i.e., ‘1’) as the difference between the first and the last answer, since long-term single episode use corresponds to a consistent use pattern. The sequential matching ensures that episodic use consists of alternating (i.e., 010101) or alternating but correlated response patterns (e.g., 0001111000001111000011). Visual inspection was performed to ensure that the patterns were matched correctly. For instance, ‘single episode users’ reflect individuals with one episode of contiguous reporting of ‘chems’ with an episode length of less than one year.
**Material S2 Interrupted Time Series Model**

The following equation was formulated and fit to each cohort dataset separately:

$$logit\left( P\left( y_{ij}=1 \right) \right)=\beta_{0}+\beta_{1}\cdot{AGE}_{ij}+\beta_{2}\cdot sex with occasional {partners}_{ij}+ \beta_{3} \cdot{cohort}_{i}+ \beta_{4} \cdot{time}_{ij}+\beta_{Lockdown and Wave 1}^{level}\cdot{level}_{Lockdown and Wave 1}+ \beta_{Lockdown and Wave 1}^{slope} \cdot{slope}_{Lockdown and Wave 1}+\beta_{Interwave}^{level}\cdot{level}_{Interwave}+ \beta_{Interwave}^{slope} \cdot{slope}_{Interwave}+\beta_{Second Wave}^{level}\cdot{level}_{Second Wave}+ \beta_{Second Wave}^{slope} \cdot{slope}_{Second Wave}+\beta_{Lockdown 2}^{level}\cdot{level}_{Lockdown 2}+ \beta_{Lockdown 2}^{slope} \cdot{slope}_{Lockdown 2}+\beta_{After Lockdown 2}^{level}\cdot{level}_{After Lockdown 2}+ \beta_{After Lockdown 2}^{slope} \cdot{slope}_{After Lockdown 2}+\beta_{First case MPOX Clade II}^{level}\cdot{level}_{First case MPOX Clade II}+ \beta_{First case MPOX Clade II}^{slope} \cdot{slope}_{First case MPOX Clade II}+\beta_{WHO PHEIC MPOX Clade II}^{level}\cdot{level}_{WHO PHEIC MPOX Clade II}+ \beta_{WHO PHEIC MPOX Clade II}^{slope} \cdot{slope}_{WHO PHEIC MPOX Clade II}+\beta_{MPOX Vaccine in CH}^{level}\cdot{level}_{MPOX Vaccine in CH}+ \beta_{MPOX Vaccine in CH}^{slope} \cdot{slope}_{MPOX Vaccine in CH}+\beta_{After Concerns}^{level}\cdot{level}_{After Concerns}+ \beta_{After Concerns}^{slope} \cdot{slope}_{After Concerns} +u_{0i}+ u_{1i}\cdot{time}_{ij}$$

Where Y_ij_ denotes the outcome of reporting chemsex substance use for subject *i* at timepoint *j*. Age was included as categories based on the difference of the year of follow-up and birth. To model abrupt/level changes from times before COVID-19, we added a binary variable if the follow-up visit fell within the corresponding period. Changes of chemsex substance use over time, i.e., the slope, were captured by continuous variables marking the time since period start. Parameters $u$ correspond to random intercept and slopes for everyone. A grid search algorithm was employed to assess lagged responses by shifting restriction periods in time. The best fit based on AIC was achieved by assuming a 7-day lagged effect of restriction periods on chems use. In sensitivity analysis, we observed a larger BIC value for the interaction model (30418) compared to the model without interaction (30246), thus, we concluded no significant morphological trend differences between the cohorts.

**Figure S2 Predictors of encountering Methamphetamine, GHB/GBL and NPS substance use in the cohorts.** Sociodemographic and behavioural predictors of each of the three ‘chems’ (methamphetamine, NPS, GHB/GBL). Results from multivariable logistic regression to discern predictors for reporting the substance at least once during the observation periods are shown in a coefficient plot. While sociodemographic and behavioural predictors are similar for the three substances, we observe different adjusted odds ratios for region and cohort.

**Figure S3 Logistic regression results after progressively extending the inclusion cut-off (by end of each calendar year) for SwissPrEPared participants**. While more participants in SwissPrEPared were recruited during the observation period, the effect of being younger than 25, and occasional having sex with occasional partners were becoming lower. The effect for participation in SwissPrEPared compared to SHCS on the adjusted odds ratios for cumulative ‘chems’ use strongly diminished over the observation period indicating that the effect being unexplained by the demographic variables (i.e., due to higher risk profiles not captured by explanatory variables) diminishes over time.

**Figure S4 Regression coefficients derived from (random intercept) GLMMs to analyse the association of the use of specific substance on specified time-updated outcomes**. In the main analysis, we compared the unadjusted and adjusted odd ratios (aOR) for substance use to predefined controls, not using any of the included substances. In the sensitivity analysis, where the definition of controls is less strict, i.e., also includes people who use other substances, we observed only minor changes in univariable analysis, however, a significant reduction in aOR in the multivariable analysis for all outcomes. The adjustment set of covariables for multivariable analysis included age, nationality, sexual preference, cohort, and year of study visit. We did not correct for nationality in the analysis of HCV infection due to convergence problems resulting from sparse data and collinearity with other variables. Univariable results remained similar, but adjusted ORs across all outcomes were strongly reduced, particularly for amphetamine and ketamine, indicating that the associations are confounded by other psychoactive substance. This was less pronounced for MDMA and cocaine. Methamphetamine showed mixed results, with a stable association with depression and syphilis but attenuated or absent associations for other outcomes. Interestingly, HCV infection showed persistent association with NPS but also for amphetamine use. These findings highlight the complexity of polysubstance use.

**Figure S5 Average prevalence of chemsex substance use (30-day left-aligned moving average)**. We calculated the average proportion and 95% (Wilson) confidence intervals of participants reporting ‘chems’ use within a 30-day moving window following each calendar day up to September 30, 2024, both that do (“yes”) and do not (“no”) report sex with non-steady partners. A peak followed by a downward slope is interpreted as a drop in average ‘chems’ use only after 30 days from the calendar date. A lagged effect is seen for the start of the first COVID-19 wave and lockdown period in the SHCS, which coincides with the start of a downward trend in ‘chems’ use rates. In the time before COVID-19, we observed significantly higher ‘chems’ use rates in SwissPrEPared, compared to the SHCS. Afterwards, reporting of ‘chems’ use remained lower in the SHCS, however, with overlapping confidence intervals indicating no significant difference within strata, after the first year of SwissPrEPared. We assumed that within cohorts there are no differences in the morphology of the trajectories, thus, we refrained from modelling the use of chemsex substance for each stratum of age and sexual behaviour with occasional partners.

**Figure S6 Comparison of regression coefficients from a generalized estimating equation (GEE) and generalized linear mixed model (GLMM) for chems use over time using and interrupted time series framework**.

**Figure S7 ‘DroMedArio’ project wastewater report data from Switzerland**. The graph shows the development of quarterly means of standardized drug metabolite concentrations (measured in seven Swiss Cantons) and prevalence in the SHCS and SwissPrEPared cohorts (summarized as ‘gbMSM in cohorts’) for methamphetamine, cocaine, amphetamine, MDMA, ketamine, and cannabis. A LOESS curve was added to highlight temporal trends. Data was downloaded from the official DroMedArio website (https://www.dromedario.ch/data, last accessed: 2025-08-28).
